# Anthropogenic, Carbon-Reinforced Soil as a Living Engineered Material

**DOI:** 10.1021/acs.chemrev.2c00399

**Published:** 2023-01-12

**Authors:** Fan Yang, Qiang Fu, Markus Antonietti

**Affiliations:** † School of Water Conservancy and Civil Engineering, Northeast Agricultural University, Harbin 150030, China; ‡ Joint Laboratory of Northeast Agricultural University and Max Planck Institute of Colloids and Interfaces (NEAU-MPICI), Harbin 150030, China; § Department of Colloid Chemistry,Max Planck Institute of Colloids and Interfaces, 14476 Potsdam, Germany

## Abstract

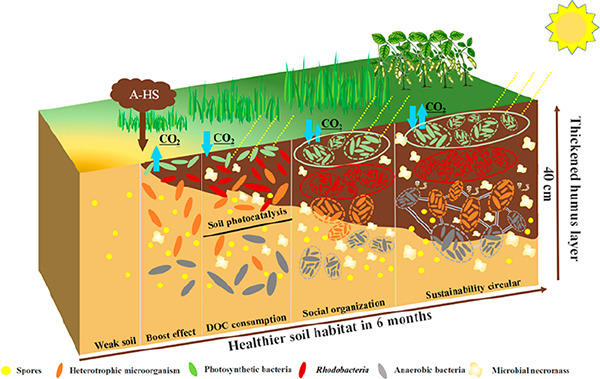

In recent years, the simple synthesis of artificial humic substances (A-HS) by alkaline hydrothermal processing of waste biomass was described. This A-HS was shown to support water and mineral binding, to change soil structure, to avoid fertilizer mineralization, and to support plant growth. Many of the observed macroscopic effects could, however, not be directly related to the minute amounts of A-HS which have been added, and an A-HS stimulated microbiome was found to be the key for understanding. In this review, we describe such anthropogenic soil in the language of the modern concept of living engineered materials and identify natural and artificial HS as the enabler to set up the interactive microbial system along the interfaces of the mineral grains. In that, old chemical concepts as surface activity, redox mediation, and pH buffering are the base of the system structure build-up and the complex self-adaptability of biological systems. The resulting chemical/biological hybrid system has the potential to address world problems as soil fertility, nutrition of a growing world population, and climate change.

## 1 Introduction: Living Materials

“Engineered Living Materials are defined as engineered materials composed of living cells that form or assemble the material itself or modulate the functional performance of the material in some manner” (https://wyss.harvard.edu/media-post/living-materials/). It is a big vision to fill the future world with such living materials, in medicine for implants and repair, for energy generation, for self-repair of constructions, or as a functional coating. Such an engineered material system mandate includes living matter and inherits as a system from the biology side attractive features as self-replication, self-regulation, self-healing, environmental responsiveness, and sustainability. To realize such a system, many disciplines have to contribute cell biology, microbiology, complex analytics, AI, and many more. A recent review^1^ illustrated the role of gene engineering and synthetic biology for biomedical applications. To complement these approaches, however, it is our tenor that old chemistry also has a strong role in this orchestra of disciplines, and here we illustrate the role of chemistry and chemically stimulated self-processes in an actual and apparently simple application, as the reconstitution of consumed soils by anthropogenic system approaches.

## 2 Soil: Its Structure and Composition

A short introduction into soil from a chemical perspective is necessarily oversimplified and focuses here on the functional properties. Soil is a living material system composed of five ingredients, that is, minerals, soil organic matter, living organisms, gas, and water (https://www.nature.com/scitable/knowledge/library/what-are-soils-67647639/). Ideal fertile soil is thereby at least a tricontinuous material with a continuous solid, liquid (including the capillary surface films of water), and gas phase. Solid minerals set the base for the mechanical stability of the soil matrix. Due to its individual aggregate form or shape structure, it is porous and the host of the other phases and ingredients, where specific surface area and pore structure are controlled by the mixture of particle geometries and sizes. The minerals are often divided by size into clay, silt, and sand. The grains are “glued” to each other toward to so-called humin–mineral complex, usually stabilizing a higher porosity than in a hypothetical pure mineral soil. In this solid matrix, an also continuous liquid aqueous phase supplies water and dissolved compounds to life, and a third continuous gas phase regulates the transport of metabolic gases. This gas phase, also known as soil air, consists mainly of nitrogen and oxygen but contains much higher concentrations of carbon dioxide and some gaseous metabolites (e.g., methane) than the outer atmosphere, which are for respiration and exchange. This triple structure is now the stage where the screenplay of life takes place.

Soil organic matter is the focus of this article. It is measured as “total organic carbon” (TOC) and “dissolved/digestible organic carbon” (DOC), and it is by its definition partly biological and living, while other parts are strictly “dead” organic molecules, polymers, and carbonaceous condensates. Historically, scientists tried to separate and isolate more specific fractions by physical extraction processes and reprecipitation,^2,3^ and the separation into humins (insoluble in acid/base), humic acids (soluble in base, insoluble in acid), and fulvic acids (soluble in acid and base) are a product of this approach.^4^ The total organic carbon content is usually between 1 wt % (used farmland) and 10 wt % (meadows).^5^ “Humus” is the latin word for soil,^6^ and obviously already the Romans knew that soil fertility depends on mostly carbon. Even in modern metrics, the amount of soil organic matter is one of the best indicators of agricultural soil quality (http://soils.usda.gov/sqi/).

Soil organic matter contains living cells and necromass from microorganisms, plants, and fauna,^7,8^ and humic matter is formed by complex abiotic and biotic chemical processes from the leftovers of living species.^9−11^ Soil microbiology, as bacteria, fungi, and archaea, is an exciting key asset in the soil system.^12,13^ A teaspoon of rich soil can contain a billion bacteria.^14,15^ There is only little known about the habits and the mutual interactions of soil microbes, as most of these organisms cannot be cultured as a single species culture in the lab. This is already a clear indication that they are living in complex, self-structured communities depending on each other, that is, many of them are specialists within a living materials system, not to be isolated from their complex matrix or the microbial neighborhoods and exchange patterns.^16−18^ New genetic tools developed to analyze the gut microbiome, however, promise help to also resolve more complex phenomena in the soil microbiome.

Chemical–biological self-processes in soil are directly observable when we dig out a soil profile: in this living system, you find self-organization and autonomous patterning, self-repair over time, and response to a changing environment, for instance throughout seasonal change. An obvious organization pattern is the layering of soils, the soil horizons. Due to the proximity of air and light, the surface horizons are most rich in life and soil organic matter, and this is which we focus on in the present article. There are, however, deeper soil layers, formed through active dynamic processes, such as mineral leaching and weathering, but also active molecular exchange between the layers.

## 3 Soils in the Anthropocene

Actually, humankind is depleting and even destroying the living matter system of soil.^19,20^ If soil carbon content is indeed the best measure for fertility, wrong farming practices and overfarming are constantly decreasing soil organic matter (SOM)^21,22^ and thereby soil fertility, especially for arable soils. Soil CO_2_ emissions are approximately 10 times higher than the emissions from fossil fuels^23^ but partly compensated by natural carbon sequestration in the soil system, which is hard to control due to the complexity of the living matter systems (in the language of this article). Consequently, research of mitigating soil CO_2_ emissions or enhancing the ability of soil living materials for carbon sequestration is apriority^24,25^ and possibly a matter of survival.^26^

If humankind can “downregulate” soil organic matter by wrong inputs into the complex matter system soil, a better understanding of the systemic processes would also allow the contrary, upregulation and recovery, with most relevant consequences for the biodiversity of farmland and forests, healthy nutrition, and a mitigated climate impact. The potential lever is enormous: the Earth offered 1.6 Gha of agricultural land and 3.2 Gha of pastureland in 2019. Any living material activity increasing the soil carbon content by 10t C per ha in fact would bind 23.8 ppm of atmospheric CO_2_, just to illustrate the potential carbon mass flux.

**Figure 1:**
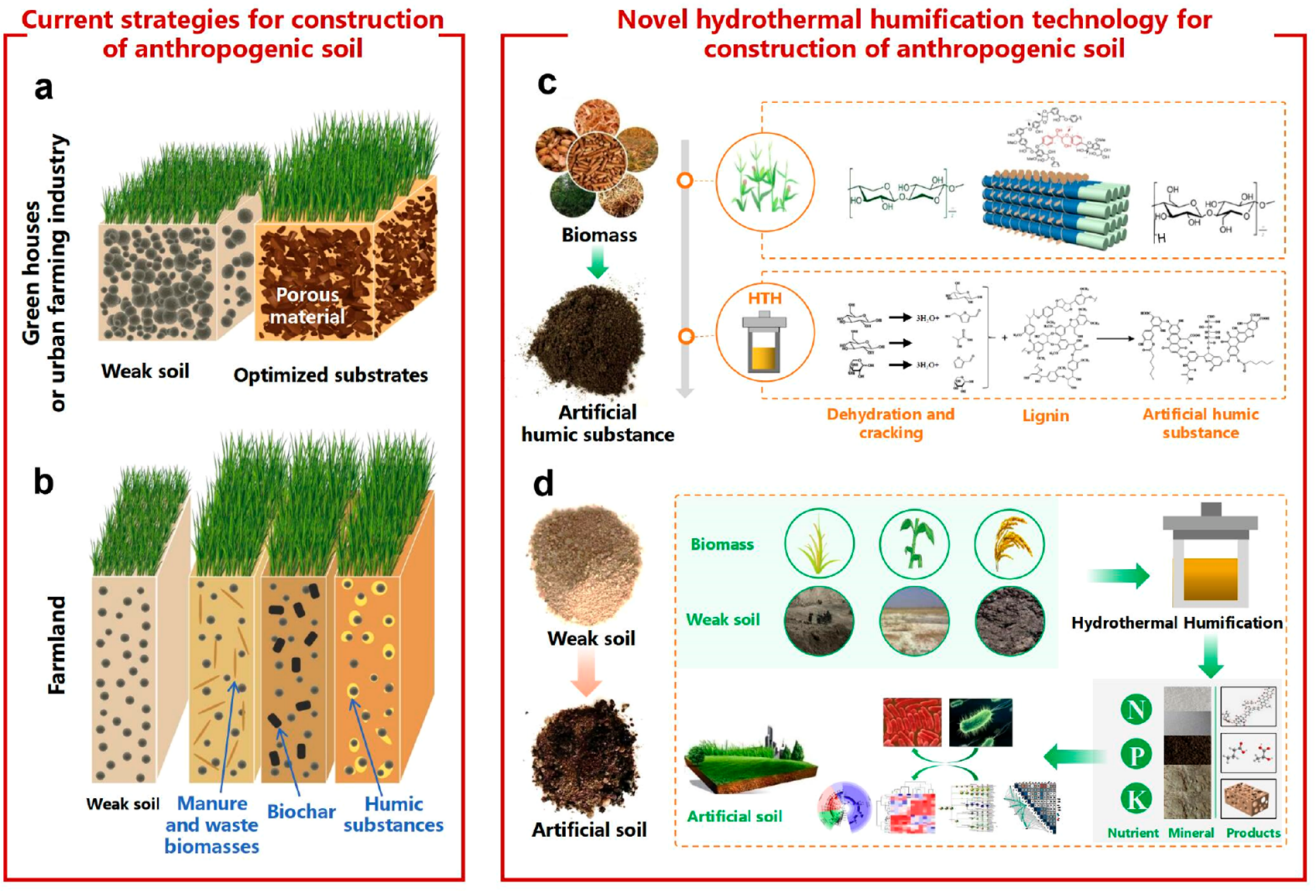
Current strategies and novel hydrothermal humification for construction of anthropogenic soil.

The concept of “anthropogenic soil systems” is not new and, in reduction and simplification, in the form of adding mined peat to otherwise carbon-poor mineral soils a tool of plant breeding in rose gardens, greenhouses, and artificial environments (Figure 1). In green houses or urban farming industry, most plants do not touch real soil anymore but grow on optimized substrates, where water and fertilizer can be continuously applied, while a porous material (e.g., blown clay) is considered sufficient to give stability. Reducing soil to such a matrix medium of course is a brute, but for greenhouses apparently feasible simplification (Figure 1a). For farmland with its alternating climate, water, and light conditions and based on the above quoted observation that sustained fertility goes strictly with soil carbon content, people can differentiate essentially three anthropogenic approaches: (i) addition of manure and waste biomasses,^27,28^ (ii) biochar addition,^29^ and (iii) natural and artificial humic substances^30^ (Figure 1b).

From our own chemical view, option (i) is, as no tilling, mulching, and crop rotation, a farming technique to improve soil quality^31^ and in that little to contribute for a chemist. Biochar is a C-rich solid formed by pyrolyzing biomass, and its addition to soil as such increases the C content.^32,33^ The preparation of biochar will partly fix the CO_2_ that plants took from the atmosphere through photosynthesis as charcoal, a believed-to-be more recalcitrant form of carbon.^33^ Expanding classical charring of dried biomass^34^ also to wet biomass and biological sludges, hydrothermal carbonization (HTC)^35,36^ is able to generate a hydrochar, also a mostly insoluble, hardly water swellable material covalently interlinked by C–C bonds in three dimensions. HTC has the advantage to work also with wet, not combustible biomass, as well as it is able to fix most of the biomass carbon in the final product,^37−40^ while charring under minor support of oxygen by principle is based on the creation of a major fraction of CO_2_ and CO. It is along newer observation that even charring does not keep such anthropogenic carbon from microbial degradation and that charring temperature, the type of soil, and conutrients are important to define a stability of biochar. In own experiments, we found a special biochar to be degraded rather significantly already on the scale of months, mostly by actinobacteria (see also discussion below^41^), when other digestible carbon sources are present. In addition, biochar addition develops its positive action on “soil material” properties only at rather high doses of addition, partly well above practical and economic feasibility.^42,43^

The candidate discussed the most in the following chapters is artificial humic substance (A-HS) made by hydrothermal humification (HTH)^44^ (Figure 1c). HTH is also using wet biomass which is obviously hard to burn, with a carbon efficiency of close to 100%, but is adding base throughout the hot water treatment. Contrary to the other techniques, it does not create solid carbon particles but rather a mixture of humins, humic acids, but also fulvic acids, i.e., most of the products are molecular, disperse very well, and act via their interface activity. This also makes the required doses for optimal activity much lower than with biochar (down to 0.3 permille weight C addition, e.g., 450 mg/kg A-HA in soil), due to the fact that a much higher number of smaller molecules is generated, which simply spread and distribute better along the mineral surfaces.

The chemical composition range of A-HS is broad and depends on starting products and process conditions. As a role of thumb, the amount of phenolic groups in the final organic condensate for instance goes with the primary phenolics/lignin input, while the number of carboxylic acids can be controlled by the added base, as every base unit can split a carbohydrate into two carboxylic acids.^44^

**Figure 2:**
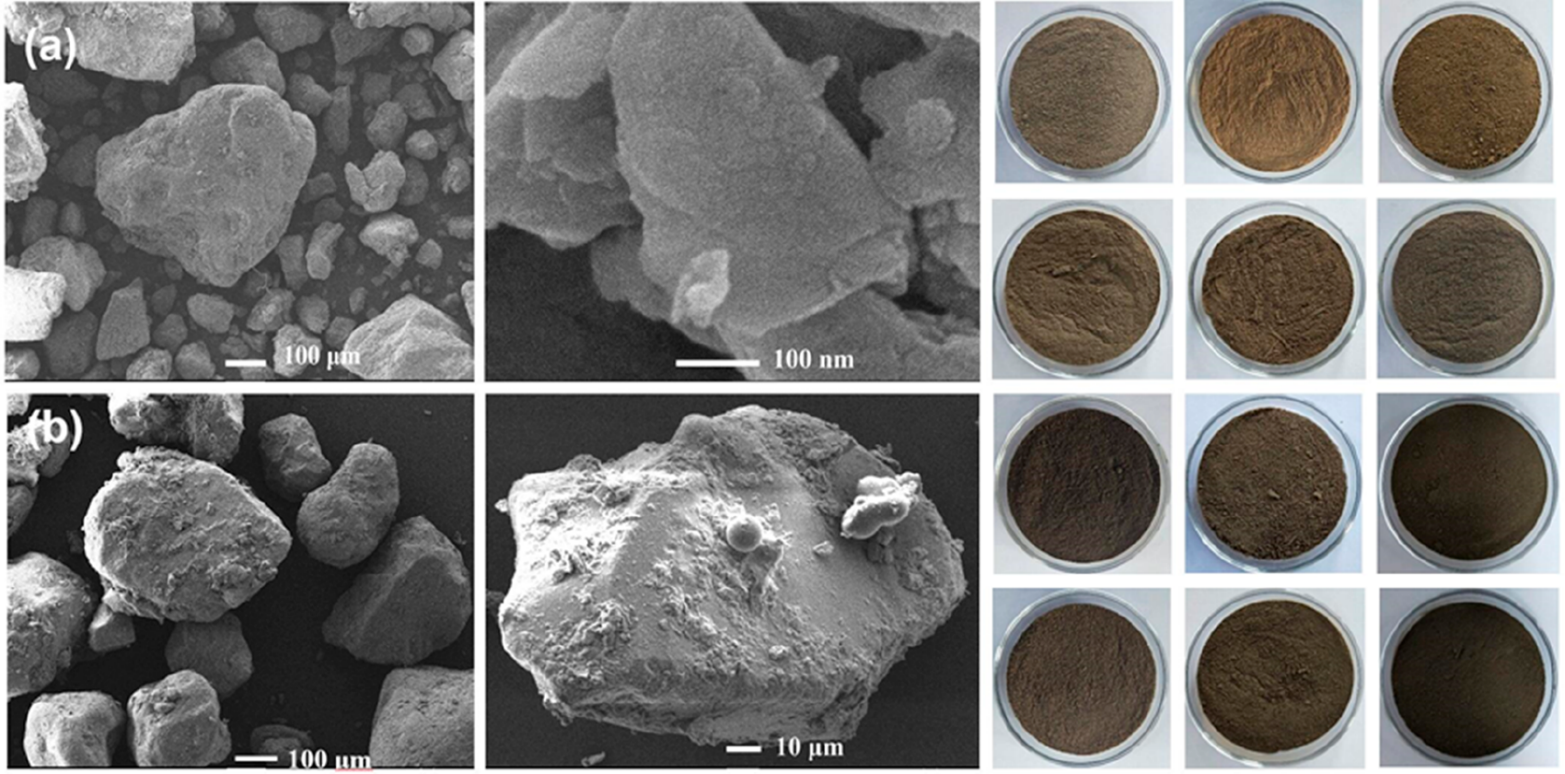
SEM images and real photos of (a) original soil and (b) A-HS amended black soil prepared through hydrothermal humification in the presence of mineral grains.^45^ Reproduced with permission from ref (45). Copyright 2020 Wiley.

A-HS can be “conjugated” to mineral soils, that is simple dispersion and mixing creates a homogeneous, with increasing amounts of A-HS, more brown-black powder which is difficult to separate again^45^ (Figure 1d). This is indeed very different from simple addition of biochar powders, which easily separate by flotation. Figure 2 shows some photographs of these products, plus corresponding SEM pictures, which illustrate that the organic material adheres to the surface as a rough film with nano- and microstructure. This is the locus where the active materials processes described in the following take place.

### 3.1 Natural and Artificial Humic Substances As a Functional Mediator Set up the Stage for Cultivating a Microbiome System

**Figure 3:**
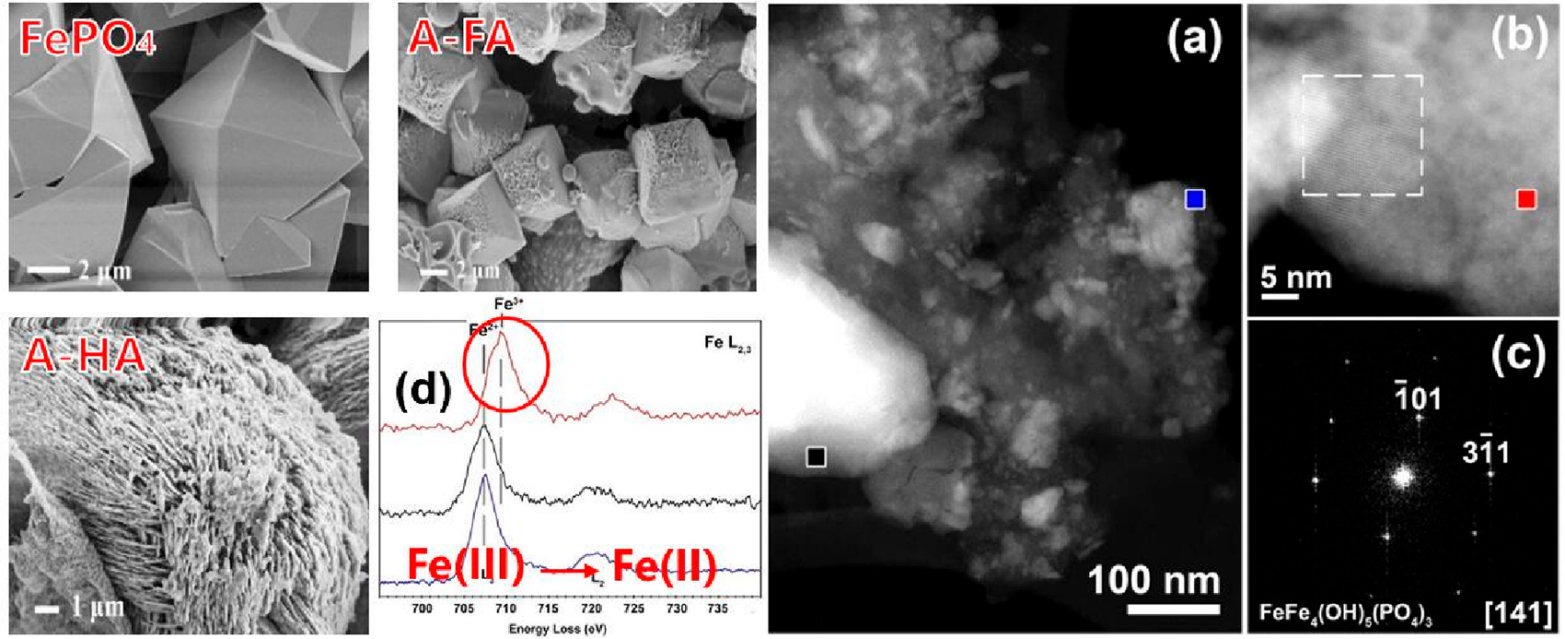
SEM images indicating the ability of humic acids and fulvic acids to actively reconstitute minerals: model crystals of original insoluble FePO_4_, reconstituted by fulvic acids (A-FA) and by humic acids (A-HA) made from leaves. (a) ADF-STEM image of nanoparticles within the “sponge” like structure. (b) HR ADF-STEM image of the crystalline particle within the “sponge”-like structure, the dotted line marks the area from which the fast Fourier transformation, presented in (c) has been taken. (d) Electron energy loss (EEL) spectra indicating that the structural changes are a reduction/dissolution and recrystallization event.^63^ Reproduced with permission from ref (63). Copyright 2019 Wiley. The whole structure change is a dynamic, purely chemical, self-organization process.

To sum up previous references,^11,46,47^ the role of humic matters (natural and artificial) as a functional additive is essentially at least 5-fold:

(1) Its surface functional groups, mostly phenolics and carboxylates,^48−50^ bind as thin layers to soil minerals, glue them together, and change soil structure toward “soft-elastic”.^51,52^ This structure also increases soil permeability and enables the buildup of the already discussed tricontinuous structure of a solid, a liquid, and a gas phase. Humic acid binds the dispersed soil particles together, and the soil forming particle agglomerate structure is branched, containing many empty interstitials, which sets the space for soil aeration and water permeability.^53^
(2) The carboxylate and phenolic groups also give humic substances the ability to bind water, but also ions either via Coulombic forces (for Na^+^, K^+^, or Mg^2+^, Ca^2+^, and Fe^2+^), metal chelation (all d-elements, especially Fe^3+^), but also by water mediated surface adsorption (e.g., phosphate).^54−56^ The partial solubility already indicates the presence of many polar groups, but they can also be ion titrated. The amount of functionality depends on origin or synthesis but can be for a typical base-soluble humic acid fraction 1 carboxylate and 1 phenolate per 600 mass units. Ion binding is important for keeping minerals in the topsoil and avoiding their rinsing to groundwater. It also immobilizes and tightly binds heavy metals by Coulombic interactions.
(3) Humic substance also contains condensed units of fatty acids and other hydrophobic lipid membrane constituents (which are for archaebacteria partly very diverse), and hydrophobic contents of up to 7 wt % in regular humic fractions have been described.^44^ Such hydrophobic cavities bind organic pollutants from water but also increase the solubility of hydrophobic metabolites and messenger molecules. Thereby, morphology, migration, transformation, toxicity, and biological effects of organic molecules and pollutants can be controlled.^57−59^ In technology, this is described as a delivery system. There are countless implications of using humic substances as delivery systems, which are partly discussed in a previous review.^11^
(4) One of the most underestimated properties of humic matter is their redox buffering capacity. Humic substances contain larger amounts of heteroaromatic and phenol-based subunits, and electrochemical experiments confirm the redox accessibility of those groups. Maurer et al. electrochemically reduced extracted natural humic matter and found a capacity of 0.54 mol protons/kg and 0.55 mol electrons/kg.^60^ For artificial humic substances, such values are in the hand of synthesis, and lignin-based artificial humic matter are most redox active. Life in general depends on a whole cascade of redox buffering molecules and redox levels, and coupling oxidative and anoxic processes in different soil domains necessarily depend on the reliable stabilization of local redox gradients also on the exocellular level.
(5) As already indicated by the very strong metal ion binding properties and the interface activity to mineral grains, humic matter also can actively change the mineral world. This is known in inorganic chemistry as the morphosynthesis of crystals or and nonclassical crystallization.^61,62^ As dynamic mineralization and remineralization processes are active in soil, humic substances do mediate between the biological and the mineral world. One of the most prominent findings is that the addition of A-HS to ordinary soils can mobilize otherwise insoluble phosphates for metabolic purposes.^63,64^ Model experiments in vitro showed that indeed apatite change to high surface area species by dynamic recrystallization in water, while the practically insoluble Fe(III)PO_4_ is reduced to a corresponding Fe(II) species (Figure 3), which is then plant available.^63^

It is to be stated that all three components of humic matter, i.e., fulvic acids, humic acids, and humic substances, contribute more or less to all of these activities, as also shown in Figure 3 for mineralization control. This is why in many experiments the crude primary mix of all species gives the broadest application spectrum.

### 3.2 From Humin Reinforced Soil to Microbe-Containing Living Materials

With all of these properties, the setting up of a tricontinuous structure, water binding, pH- and redox buffering, mineral interactions, delivery, and detoxification, the added carbon species are ready to support life. It is a fact that there is only very little life at very low TOC, say in sands, and TOC is also quantitatively still the best indicator for soil fertility.^65,66^ Meadow soils can accumulate up to 10 wt % of carbons and are among the most fertile soils known^67^ but take up to 3000 years to build up naturally.

However, when the presence or addition of humins is excessive, it can cause harm. As it contains a large amount of soluble substances, high dose humic substance will create a high osmotic pressure and has a certain inhibitory effect on growth and reproduction. Excessive acidic pH will inhibit the proliferation of microorganisms such as actinomycetes. The abundance of microorganisms and the activity of enzymes in the soil at such carbon levels is then declining again. Pure peat or brown coal are not effective substrates, setting the final point.^68^

### 3.3 The Different Phases of Microbial Soil Colonialization and How to Address Them with Anthropogenic Carbon Sources

**Figure 4:**
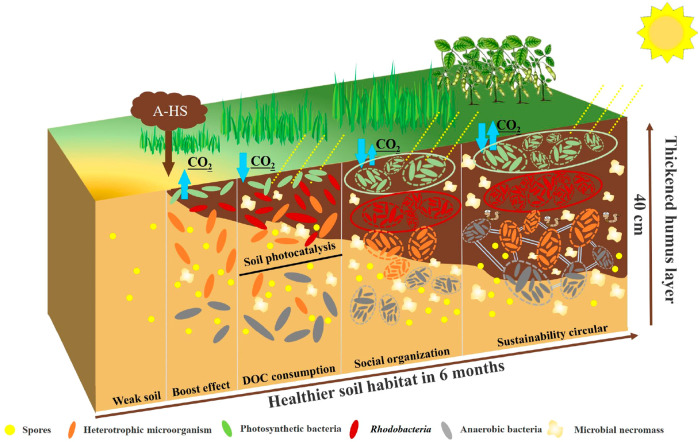
Schematic sketch of an original weak soil, A-HS addition, and the following phases “boost growth–DOC decrease/soil photosynthesis–social organization–sustainability circular” to reconstruct a more healthy soil system. For simplicity, only interactions with microbiota are discussed.

When adding A-HS to soils, there is a cascade of processes observed, and scientists are just in the first stages of follow ing the development of complexity. As illustrated in Figure 4, we simplistically divide the observed phenomena in 5 phases, which all occur more or less depending on the soil conditions and the composition of the added humic substances. We also underline that the reviewed model experiments were done in the absence of higher plants, that is, the described dynamics are, as far as possible, only due to the soil microbiome.

(a) In the first chemotrophic phase, a “***boost***” or “nucleation” effect can be generated. Microbes rapidly grow, presumably on the expense of the added carbon compounds. It was experimentally found that the proportion of *Proteobacteria* and *Actinobacteria* after A-HS addition is 1.31 times and 3.43 times higher than those in the control group after only 7-day cultivation at room temperature, suggesting that A-HS indeed greatly increased the relative proportion of soil microorganisms.^69^ In this case, a substantial amount of low molecular weight organic acids contained, e.g., as acetic, lactic, or in general fulvic acid, can simply be considered as an effective metabolite to feed microorganisms.^70^

(b) After the nucleation of this chemotrophic phase, we have to differentiate between the absence and presence of light. Under the action of added exogenous carbon and no light, total and *dissolved organic carbon* in the soil system *decrease* on the monthly scale (in the cited experiments 45 and 90 days).^41^ This is the direct product of the high metabolic activity boosted before. To our surprise, microbial life in this phase is rather resilient, even throughout winter and frost events, as it will be presented in detail below. Dissolved organic molecules and salts decrease the melting point of water as well as the structure of water crystals, and life indeed might be kept active in high concentration organic carbon droplets in otherwise frozen soil.

In the presence of light, autotrophic species and species with the ability of multiple possible metabolic pathways take over (e.g., *Rubrivivax gelatinosus*, which can switch from chemotroph to anoxic-photosynthetic to autotroph). This starts in the presence of light a phase of “*soil photosynthesis*”, which is extremely efficient. The chemical action of added A-HS is not in the first line, only bare addition of carbon by chemical or physical means, but activates the microbiome, and the resulting biological amplification can bind a multiple of the original added carbon.

**Figure 5:**
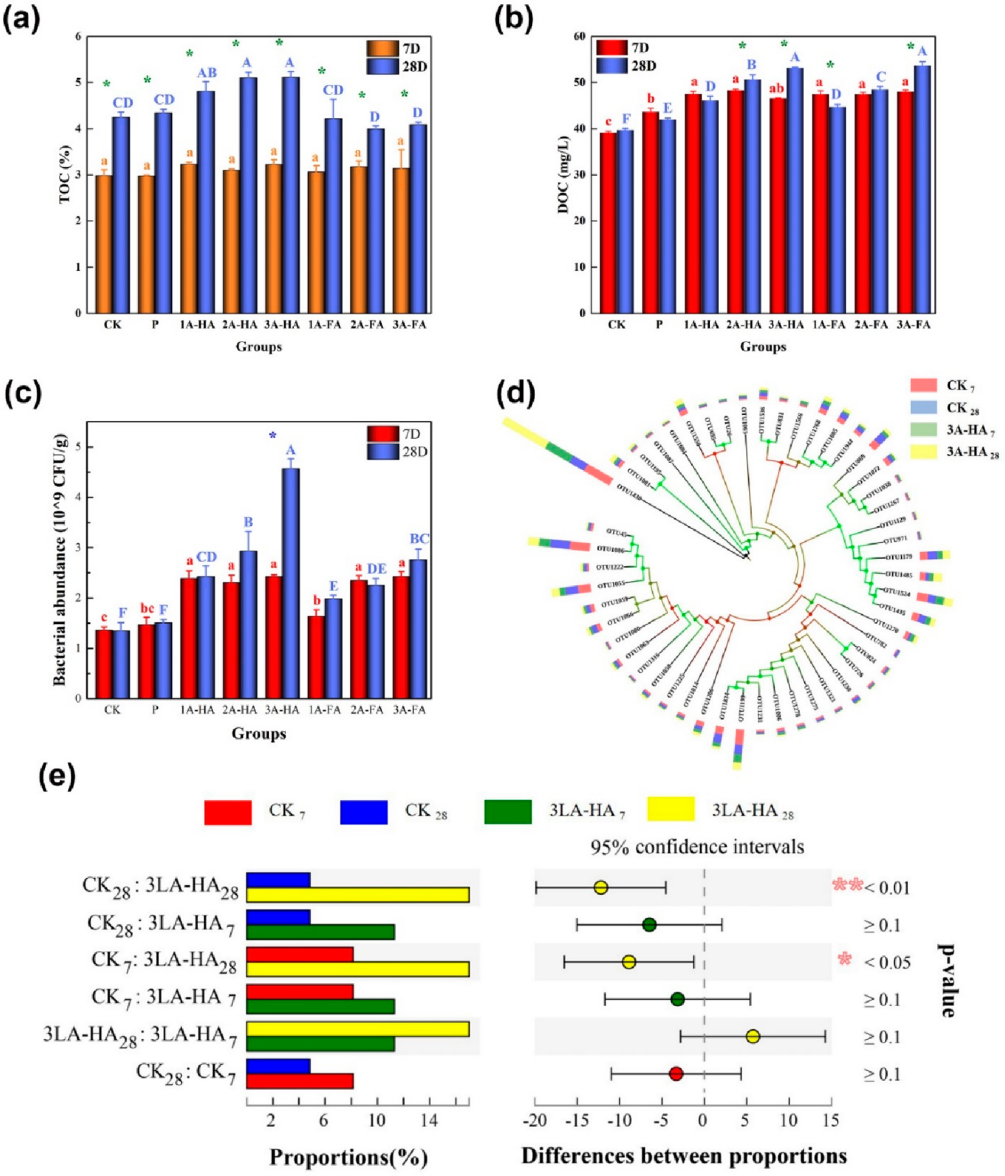
(a) TOC and (b) DOC content of soils treated with different doses and types of A-HS and light after 7 days and 28 days. DOC only slightly increases, while the total carbon partly massive increases. (c) The abundance of bacteria as revealed by high throughput sequencing. (d) The community composition and taxonomic information on dominant soil carbon sequestration bacterial with *cbbL* genes. (e) The posthoc test of the relative proportions of OTU1430 between the most effective treatments and the control group. CK indicates that there was no addition measures and 3LA-HA indicates that 450 mg/kg artificial humic acid was applied to the soil, and subscript numbers represent the period of cultivation.^69^ Reproduced with permission from ref (69). Copyright 2021 Elsevier.

In a recent paper, our group described the influence of the addition of up to only 0.45 wt % of A-HA under light conditions^69^ (Figure 5) and analyzed the influence on typical soil parameters of an already strong agricultural black soil from Harbin, China. Instead, really seeing the partial metabolization and degradation, we found the opposite. As shown by the data, total organic carbon content of the soil increased by up to 2.1 wt % (compared to the added 0.03 wt % C). We could show that this increase was proportional to A-HA addition, and the light shone on the soil sample. Metagenomic analysis of the bacteria grown in the soil allowed identification of *Rubrivivax gelatinosus* (a photosynthetic rhodobacterium), as well as a carboxydotrophic bacteria which can oxidize CO and fix CO_2_ through the Calvin–Benson cycle^71^ as the main primary species to explain the extra carbon bound from the soil air. *Rubrivivax gelatinosus* is an archaic, pre-plant age workhorse in C sequestration,^72,73^ and the addition of A-HA obviously supports a rich bacterial community based on the primary *Rubrivivax gelatinosus* activity. This is literally an explosion of the living matter system. As seen from Figure 4, the gained carbon is not only in living microbes, but mostly already freshly synthesized chemical carbon compounds accumulated from a number of lifecycles of bacterial necromass, that is, also freshly formed natural humic substances, as identified by fluorescence spectroscopy on the soils. Integrating all these processes, A-HS “catalyzed” the formation of significantly more natural humic substances, that is, the details of the chemistry of the primary compounds loose relative importance when analyzing soil carbon later. Such observations also relativize ^14^C isotope experiments, as primary carbon added and secondary carbon bound complicate simple sum analyses.

The importance of “soil photocatalysis”, that is, efficient photochemical CO_2_ binding by soil microbia, is not new but was already observed in a number of elder experiments, there on biochar. It was validated that the addition of biochar increases Rubisco activity,^74^ the most important enzyme to control and mark microbial CO_2_ sequestration. In a further, detailed study, the regulation of microbe community structure by biochar was analyzed. A higher addition of biochar to paddy soil pronouncedly increased the abundance of C sequestration genes, involved in Calvin–Benson cycle, 3-hydroxypropionate cycle, and 4-hydroxybutyrate cycle, separately, as identified by quantitative real-time polymerase chain reaction (qPCR).^75^ Redundancy analysis revealed that the redox potential, the C/N ratio of input fertilizer, and NO_3_^–^-N content had significant influence on the abundance of CO_2_ sequestering microbes.

(c) After the phases of simple food and establishing the primary colony, a phase starts where self-organization and social colony formation takes place in the connected adlayers of the soil grains stabilized by A-HS. This is less seen in changes of the TOC but in tube experiments (see below), and the increment of microbial life does not necessarily increase overall C content, as the balance between C loss and C increment by microbial activity is now fully active. We might simplify the ongoing processes that the Darwinistic stress (e.g., by seasonal climate changes or declining simple food sources) now faces microbial life either to decline or into ***social organization***. Here, anthropogenic carbon-addition has the potential to change the development of the screenplay, and the community structure and microbial abundance in dependence of anthopogenic interactions are key parameters to be analyzed.

Indeed, the diversity of soil responses point to the fact that the experiment is more complex than only “adding biogenic carbon”. Some investigations revealed that biochar amendment on soil reduced soil heterotrophic respiration strikingly, resulting in a decreased C degrading microbial activity.^74^ On the other side, Steinbeiss et.al^34^ investigated that yeast-derived and glucose-derived hydrochar added to soil even increased the respiration rate of soil microbes. Ye et al.^76^ described the variation of the diversity between bacterial communities on the surfaces of one type of biochar and two different mineral-enriched biochars after 140 days of incubation in soil. Chemolithotrophic bacteria with the capacity of sequestrating additional CO_2_ were found dominant upon the surface of biochar, which locally translates to our reading into a process where biochar is used as an energy source for CO_2_ refixation, i.e., it can be described as a bioelectrochemical conproportionation process. Xu et.al^77^ analyzed the reasons for the diversity of reactions of soil’s heterotrophic respiration to biochar and confirmed that biochar properties and amounts, climate conditions, exposure methods, and time do matter.

Adding the right type of carbon source with the right properties at this point of the development can be regarded as the key step of successful living material formation. It comes with slowed down further growth but dynamic stability, self-adaption, and resilience. As we know from ancient anthropogenic soil systems, the as- created system is then, however, good to survive partly for thousands of years (e.g., so-called “Machair” from Scotland^78^).

As an example for the involved colonial organization processes, we can discuss the possible nitrogen fixation from the atmosphere which turns into a key restriction to solve at this stage after bacterial explosion to allow further growth. “Townships” of nitrobacteria have to be supported by other townships of autotrophic or chemotrophic species, both of course being in separated locations, as the first prefer an anoxic lifestyle, while the second are oxidic.

Such organization is impossible without stabilizing the corresponding redox gradients on a materials level, that is, the above-mentioned “right type of carbon”. Humic substances as redox buffers are providing the chemical base to build up such spatially organized redox gradients, and presumably other bacterial specialists are active to establish and keep them. As obviously healthy soil contain at various places both oxidic (e.g., soil photosynthesis) and anoxic processes (e.g., ammonia or methane generation), the presence of stable redox gradients and operation domains is multivarious. There is still little known in soil on such structures, but we might learn from freshwater sediments where gradient formation was analyzed in more detail. Kappler et al.^79^ analyzed electron shuttling via humic acids in the lake Constance and found mostly Fe(III) in the aerobic top layers, where Fe(II) was dominant in the reductive, anaerobic lower layers. When analyzing the bacterial polytype, they found substantially larger populations of humic acid reducing bacteria than iron reducing bacteria, with HA based bacteria being even on the same number scale than the chemotrophic, fermenting bacteria. This gives strong evidence that indeed humic substances are the main biologically active redox buffer and that bacterial community massively invests in specialists to organize redox gradients and structures. We assume based on the data that it is the redox activity of the added A-HS which makes the difference between rise and fall of the whole system.

(d) The ***phase of sustainability*** of microbial soil colonies is maybe the key for the observation that anthropogenic soils of ancient cultures after calculated hundred thousands of lifecycles of microbes are still more fertile than the soils in their environments (for examples as terra preta or Machair). Obviously, in this phase, the spatiotemporal organization and layering allows an efficiency of the living matter system being a discriminative, evolutionary advantage. It can for instance be speculated that CO_2_ generated by metabolization is buffered in the soil and used in the upper layers for photosynthesis, which as such in return creates the organic matter to be degraded when water-sedimenting into the lower layers.

It is one of the most interesting observations that as a consequence of microbiome organization further down the timeline, CH_4_ emissions can be lowered by the addition of anthropogenic carbon, too. This is from a chemistry view no direct surprise, as methane is a potential high energy hydrogen source (“food”), and release of methane from an effective microbiome system an unwanted leak of carbon and especially electrons. As a consequence, there is usually in natural systems a fierce competition between microbial CH_4_ generation and respiration under anaerobic conditions.^80^ In general, microorganisms prefer to get electron–proton pairs from microbial oxidation of organic substrates (as methane), as long as there is sufficient digestible organic matter in the soil.^81^ Ye et al.^82^ reported that the presence of humic substances provides a more efficient electron acceptor within the anaerobic respiration in fen soils and directly inhibits the emissions of CH_4_. Although the phenomenon that HS inhibits methane emissions is common to all wetlands, the effects of soil composition on methanogens can be diverse.^80^ Cervantes et al.^80^ proved the key role of redox buffering by introduction of anthraquinone-2,6-disulfonate (a chemical model redox buffer) in the methanol–methanogen system and successfully reduced the CH_4_ generation by inhibiting acetoclastic methanogens. Similarly, the investigation of Ye et al.^82^ displayed that humic substance addition to bog soils inhibited the production of CH_4_ by 86% owing to its inhibitive action on methanogens. Also, Keller et.al^83^ described that humic substances inhibit CH_4_ emission in wetlands. Using biochar as anthropogenic carbon, Wang et al.^84^ proved that addition to paddy soils reduced the emissions of CH_4_ in a four-year study. Further, he clarified the role of the microbial community structure, and the added biochar significantly suppressed the abundance of methanogens, while having less impact on the abundance and activity of methanotrophs. We assume that these observations can be generalized to most metabolic substrates being locally recycled for competitive system optimization.

### 3.4 A Four “Season” Track Experiment

These different phases motivated us to start a comparative, accelerated “4 seasons in 180 days experiment” where anthropogenic modified soils with different amounts of A-HS as well as AHS–biochar mixtures in comparison to the reference samples were analyzed.^41^ In these experiments, the amounts of carbon input derived from different A-HS doses were in the range from 332.42 to 1994.52 mg/kg (0.03–0.2 wt %), respectively. The carbon input derived from BC was up to 12500 mg/kg (1.25 wt %). The experiment was also ment to compare the performance of biochar and A-HS, with a typical dose difference of a factor 10 as known from many previous experiments.

**Figure 6:**
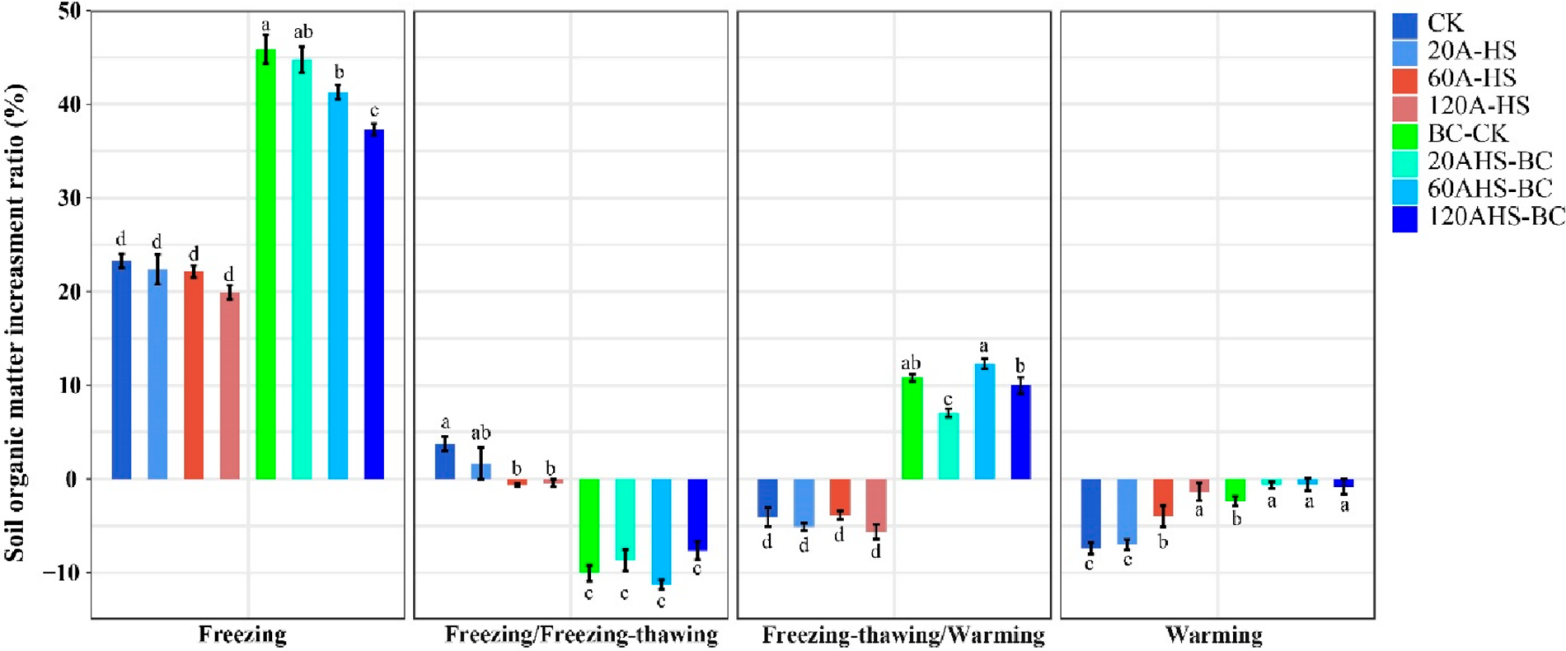
Relative increase and decline of soil organic matter in a “seasonal” sequence, separated in rough boxes called early winter, winter, spring, and early summer (year 2021–year 2022). The letters a, b, c, and d indicate the variability within experimental groups. Error bars present standard errors of experimental repeats.^41^ Reproduced with permission from ref (41). Copyright 2023 Wiley.

Some of the findings are displayed in Figure 6. The difference in SOM between adjacent cultivation periods (ΔSOM) for control group and different experimental compositions shows indeed most of the discussed effects. In “early winter before frost”, when the carbon was freshly added, the SOM content did increase but significantly less than expected by addition. For instance, real SOM increase for 120AHS-BC treatment (the combination of 120 mL/kg A-HS solution and 2 wt % BC) was 37.29%, while the calculated value by addition should be 58.29%, all relative to the original carbon content). Comparing with the control group (and low carbon doses), a large quantity of carbon is indeed metabolized (the more with more A-HS) is added, and particularly biochar is quite quickly metabolized (a clear assignment already through the added amount). This reflects the “boost” phase, and the activated soil system starts to set up its own equilibria by metabolizing biochar, ignoring the simple intention of humankind to store carbon.

In the second phase (winter), the samples underwent machine-made freeze–thawing events, and as compared to the control group, metabolic activity under A-HS addition is kept high even in the frost phase, and the carbon content is quickly declining further. This leads even to a negative value of soil organic matter increment, backed by the needs of a big bacterial community to eat and stay alive.

In the warming season (spring), the introduction of A-HS led to continued SOM loss during BC addition, while the combined application of A-HS and biochar made the TOC increase again, largely due to immediate soil photocatalysis. In the sight of the biochemical reactions in soil advancing soil carbon sequestration, several major microbial carbon sequestration pathways, such as the Calvin–Benson cycle, can be activated to bind carbon, all of that driven primarily by photosynthetic bacteria but partly also by chemoautotrophic bacteria.

**Figure 7:**
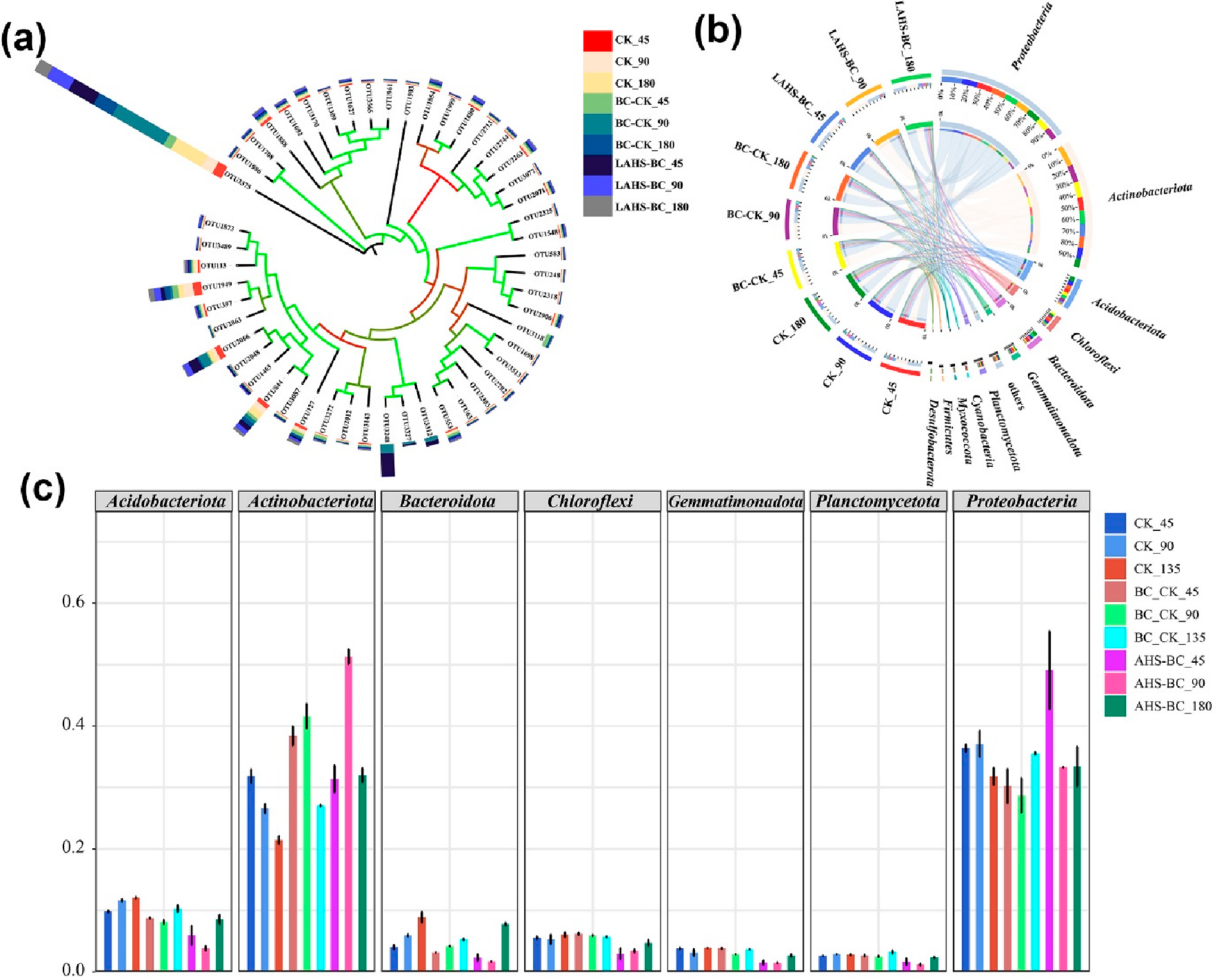
A series of high-throughput sequencing results. (a) The evolutionary tree, along with relative abundance of OTUs. (b) Circos plot at the phylum level. (c) The ratio of each phylum accounting for the bacteria composition in different soil samples treated with different carbon addition schemes. Letters stand for the diverse carbon species and control groups, numbers indicate the duration of the experiment.^41^ Reproduced with permission from ref (41). Copyright 2023 Wiley.

On the biological side, the regulation of the bacterial community structure and composition by combined application of (A-HS) and biochar (BC) can be followed during seasonal climate changes by high-throughput sequencing. For example, at 97% sequence similarity level, operational taxonomic units (OTU) numbers in the experimental groups varied from 2107 to 2644. The soil sample with treatment of a combination of A-HS and BC (AHS-BC group) after 45 days of cultivation had the lowest width of OTU (indicating the taking-over of a few driving species), while on the contrary, the diversity of OTU in the original soil (control group) after a 180-day cultivation period (freezing–freezing–thawing–warming condition) was the largest (Figure 7).^41^

In the sight of bacterial community composition at the OTU level (Figure 7a,b), we can specifically follow the function of A-HS. For instance, the relative abundance of OTU 3375 (*Actinobacteriota*, which have the ability to degrade soil organic matters) varies significantly among CK, BC-CK, and AHS-BC groups. In detail, the relative abundance of OTU 3375 in the 120 AHS-BC treatment was conspicuously higher than that in the BC-CK treatment (*P* < 0.05), followed by the control CK group (*P* < 0.05). In simple words, A-HS creates the fermenting conditions in soil supporting the after-frost activities of species described by OTU 3375.

On the basis of the “clusters of orthologous groups function classification” (COG) results, the abundance of the part of COG part associated with metabolism was related to the different treatments and cultivation periods. The observations clearly show the massive increase of factors associated with amino acid and carbohydrate metabolism for both BC and AHS-BC treatments in winter, while the control group stays frozen (*P* < 0.05). After the 180-day cultivation period, the abundance of virtually all metabolism-related COGs drops significantly again (*P* < 0.05). Notably, the results of fluorescence spectra on dissolved organic matter also show that the highest fluorescent indices (FI) and biological indices (BIX) appeared in the experimental group containing higher doses of A-HS at the early incubation stage, which demonstrates that the addition of A-HS stimulates microbial growth followed by the increased production of highly condensed (fluorescent) microbial metabolites.

### 3.5 Tube Experiments in the “Soilarium” to Identify Complex Space-Temporal Organization Patterns

To illustrate spatial organization and patch formation in soil with a horizon stacking of up to 40 cm depth, but also lateral coorganization, a tube experiment (10 cm wide, 60 cm high), similar to the traditional Winogradski column (https://en.wikipedia.org/wiki/Winogradsky_column) was designed to have a direct look onto the developing soil system. The system can be shadowed with a sheath, and admittedly, it is more an educational illustration than a perfect model of the real situation, as surface effects along the plastic tube cannot be excluded. This is similar to a terrarium, but as no higher animals are involved, we tend to call it a “Soilarium”. Of course, watering, light, and soil composition can be changed and tested, and samples can be taken with a metal probe. Water permeation can be measured by transmissive flux rate, water uptake by weight gain, and many soil parameters are simply accessed in a simple, single laymen experiment. Most importantly, the highly accelerated development of a microbiome can be nicely observed in the direct comparison of tubes with and without anthropogenic carbon or with other Winogradski columns in the literature.

**Figure 8:**
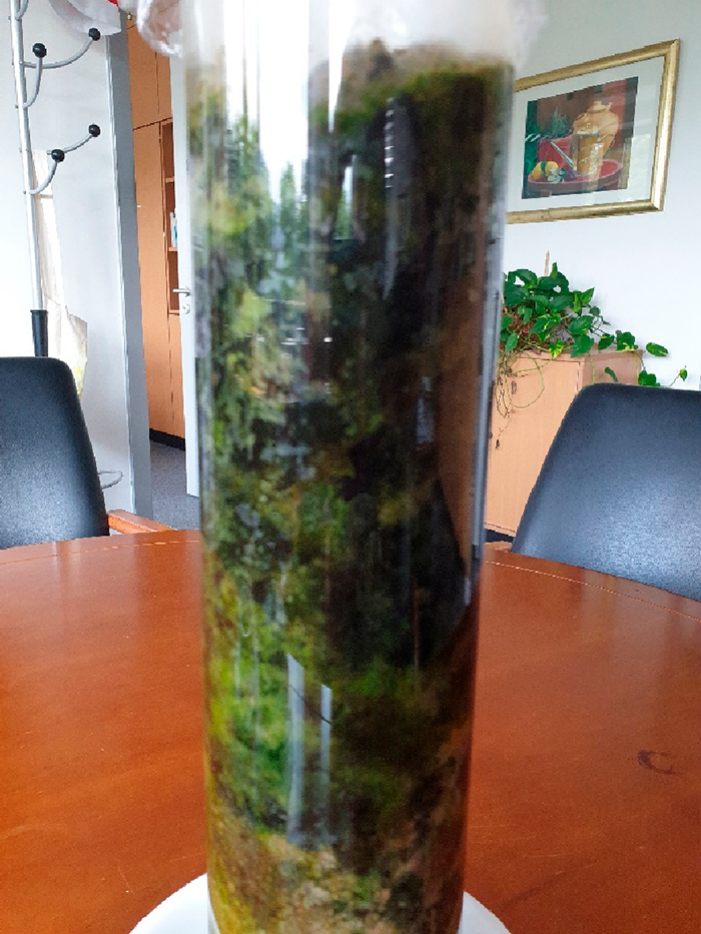
Photograph of a tubular “soilarium” after 3 months of cultivation under window daylight, water given once a week. The special self-organization in colonies, green, dark-red, and black layers and patches, is clearly observable, with a lateral structure size of the black patches of 1–3 cm.

One exemplary tube with daily room light contact over the complete tube 3 months after seeding the tube is shown in Figure 8.

One can nicely depict the role of green and red soil photosynthesis, as well as the later formation of black, potentially anaerobic bacterial patches. Colony formation and structuration takes place on the cm scale in all dimensions.

## 4 Applications

### 4.1 Seedling Soil of a New Type, Containing Plant Supporting Microbia

Growth of seedlings from seeds is a standard greenhouse practice and a model case where modern technologies can be tested in a very controlled and environmentally noninvasive fashion. In a recent publication,^85^ the case of maize seedlings was chosen, and the standard agricultural practice of adding phosphate fertilizer was compared with the new case of adding phosphate and A-HS at the same time. Here, the rationale was not focusing on the microbiome but rather on increasing the availability of phosphate also be chemical and physical means.

**Figure 9:**
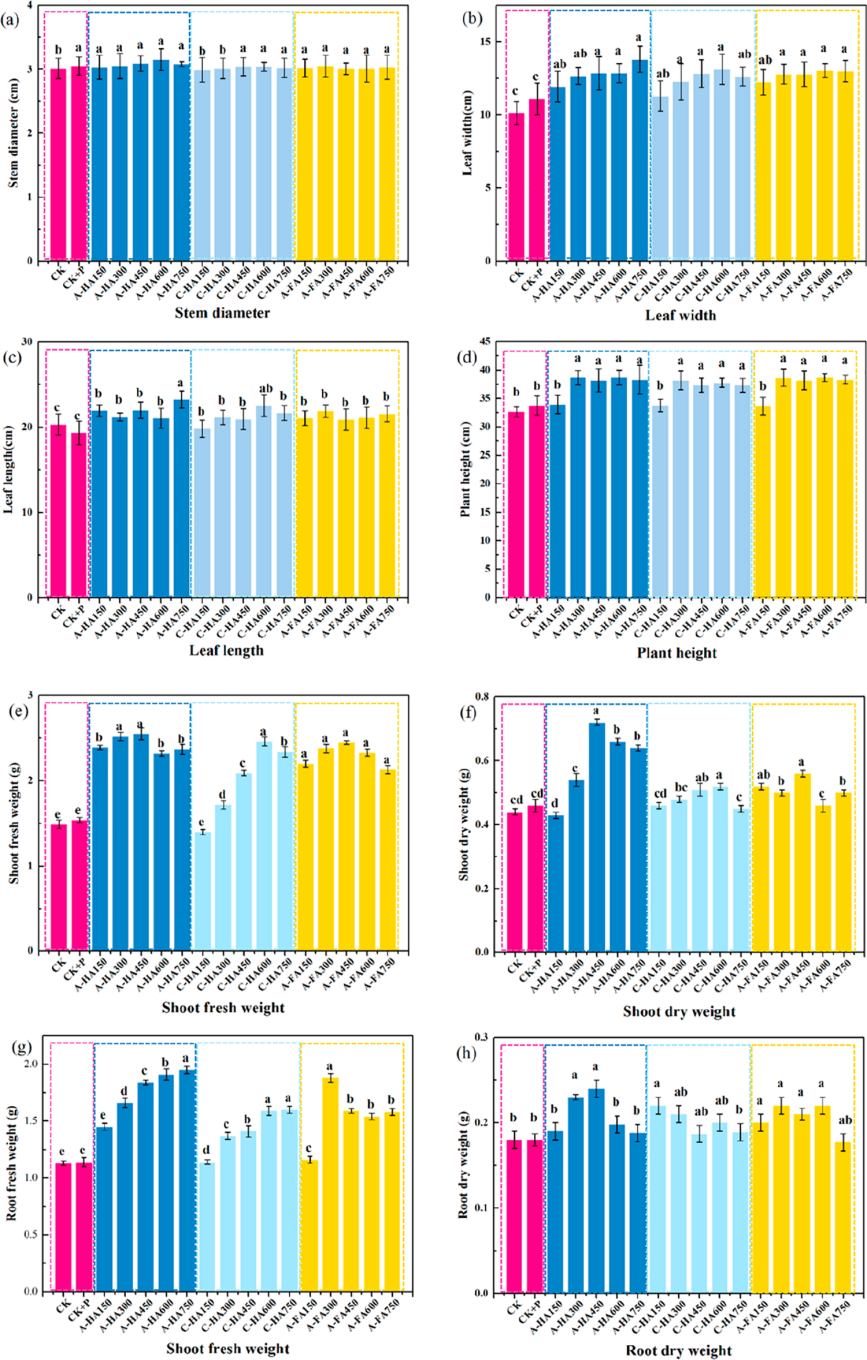
(a) Stem diameter, (b) leaf area, (c) leaf length, and (d) plant height, (e) shoot fresh weight, (f) shoot dry weight, (g) root fresh weight, and (h) root dry weight of maize seedlings. The letters a, b, c, and d mark behavior groups with significant differences at *p* < 0.05. Treatments marked with the same letter behave similarly but remarkably different to the other letters. Error bars present standard errors of experimental repeats.^85^ Reproduced with permission from ref (85). Copyright 2022 Elsevier.

Application of A-HS and phosphate fertilizers significantly promotes the growth of maize seedlings, as reflected in practically all plant growth parameters, as depicted in Figure 9. Phosphate fertilizer applied to the soil alone (CK+P) observably promoted stem diameter and shoot fresh weight, but did not reach significant improvement levels for leaf area, plant height, shoot dry weight and root dry weight. Comparing the three analyzed humic substances, the dry weight under A-HS treatment at the same added concentration was markedly higher (*p* < 0.05) than that under commercial leonardite-extracted humic acid and A-FA treated samples, and its growth-promoting effect was larger. The type and concentration of A-HS also affect the morphological indicators of the seedings, with A-HA having a more pronounced effect on plant morphological indicators than A-FA as well as commercial HA.

### 4.2 Soil Priming

**Figure 10:**
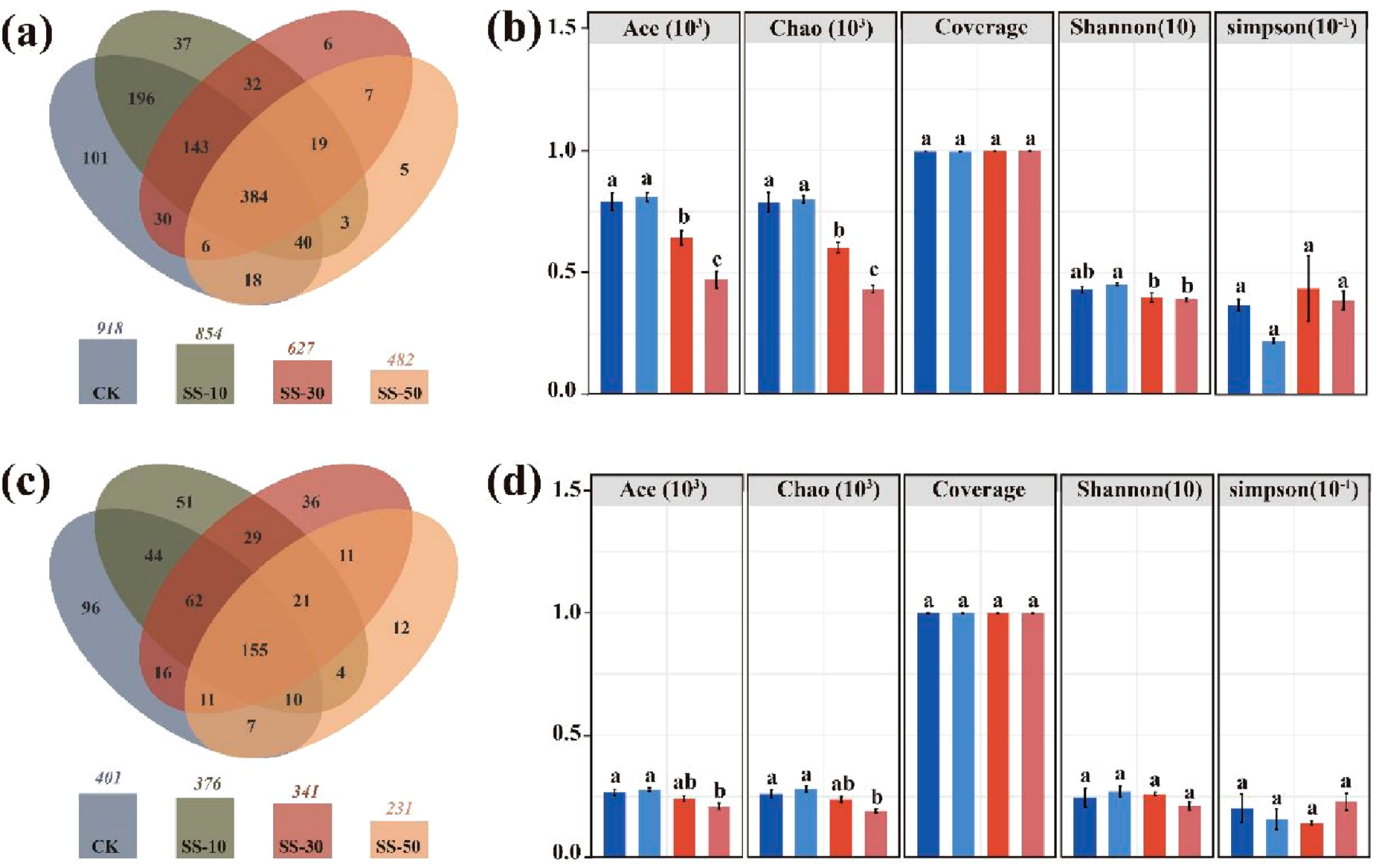
Veen and species composition diversity of bacteria (a) and fungi (c) of original soil and the artificial soil constructed by the mixture of black soil derived from hydrothermal humification reaction with the original soil in ratios of 0%, 10%, 30%, and 50%, which are noted as CK, SS-10, SS-30, and SS-50, after 30 days postconstruction. (b,d) Ace and Chao index are two measures of the richness of species; the larger the value, the higher the richness. Shannon and Simpson index are two measures of the species diversity. For Shannon, the larger the result, the higher the species diversity; for Simpson, the smaller the result, the higher the species diversity. Coverage indices of sequencing were >0.999, indicating that the sequencing results indeed represent the real situation of the species and structure of bacterial community.^86^

The next step of the anthropogenesis of soil as a living matter system is the priming of natural, poor soil with minor amounts of a reinforced artificial soil. The rationale behind such an experiment is that the newly established optimized anthropogenic soil material system contains all relevant chemical molecules, but also all grown microbial species to reorganize after mixing on a larger scale, i.e., the primary living matter system can act as a “primer” and could be “diluted” (Figure 10).^86^

It is clear that the mixing will destroy the former established spatiotemporal organization patterns, but the bacteria provided might be able after “plugging” to reactivate organization in the then diluted state. This mostly relies on the fact that the added humic matter supports resocialization.

In the Veen plot of Figure 10, we indeed can learn that most of the bacterial species, but also fungal species, stay observable. Indeed, many species are, however, rather typical in weak soils and apparently not visible anymore in strong soils, with only a few species being dominant in strong soils. The experiments show that dilution of up to a factor of 3 seems to be uncritical. Such data, however, must be complemented with other enzymatic and genomic tools, as exactly identification of those species being in the rim regions of the Veen plot carry the information on the changing social organization.

### 4.3 Carbon Sequestration

**Figure 11:**
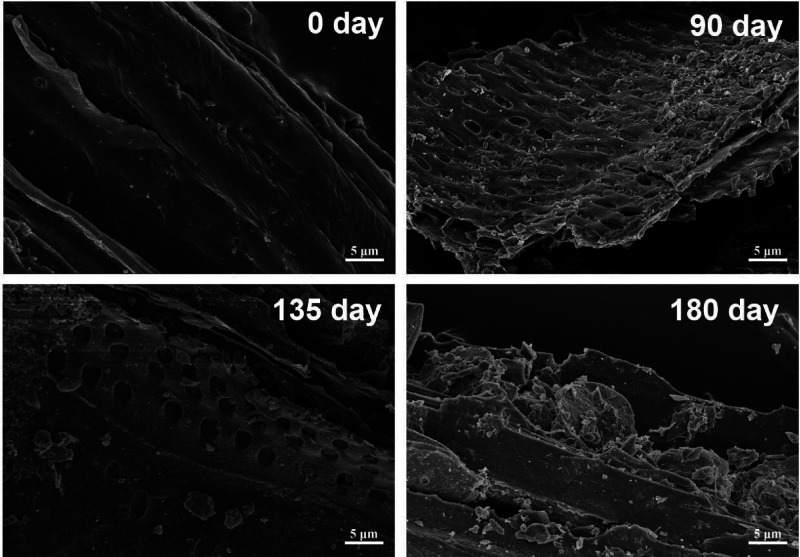
SEM images of original biochar and aged biochar collected from soil experiencing 90-day, 135-day, and 180-day cultivation.^41^ Reproduced with permission from ref (41). Copyright 2023 Wiley.

The more early experiments and reports of adding anthropogenic carbon to soils were motivated by the binding of carbon in soil for climate remediation, and the first drop by immediate metabolization of accessible parts was taken as a disappointment, as many scientists linearly progress the rate of decline to determine a “lifetime” of soil carbon.^46,87,88^ The above-described experiments of “4 seasons in 180 days” already quantified via total carbon content how fast original anthropogenic carbon in fact can be metabolized, the presence of an appropriate eager microbiome and a conutrient assumed. We also already discussed the composition of the microbiome stimulated under such conditions and at least plausibilized actinobacteriota as the players degrading carbon into elsewhere needed, active, organic molecular compounds. The experiments interestingly allow to reisolate the carbon from the cultivated samples and follow the altered morphology of at least biochar (note that A-HS are mostly aggregating molecules and can dissolve, change shape, or spread onto surfaces) on the length scales of the diverse microscopies. This is shown in Figure 11.^41^

Indeed, one can observe how a carbonized sheet-like plant fragment (here from chopped corn stalks) is peeled of its outer layers already after 90 days in a fulvic acid supported microbiome system. The 135 days reveal already the inner support structure of plant lignification, while after 180 days, only broken fragments are left. Our human intentions of carbon fixation obviously turned out to be considerably oversimplified, and there is in our opinion no direct justification to ask for carbon credits and CO_2_ certificates based on the added amount of carbon to soil.

Whenever first approaches turned to be naïve or just too linear, Nature puts usually more beauty in the unexpected answer. (“Part of being successful is about asking question and listening to the answers”, a common quote of Anne Burrell).

Obviously, we reported in the paragraphs above a partly massive increase of TOC in correctly formulated chemical soil systems, while at the same time significant parts of the anthropogenic carbon are metabolized within the different phases of microbial colony formation. This is not a contradiction, but a clue. We consider this observation as rather typical for a living hybrid material; the carbon is not stored in individual compounds to follow, but on the system level, and such systems in the sustainable phase can easily survive thousands of years, as seen with the ancient cases of anthropogenic soil systems. Added anthropogenic carbons vanishes, as it is digestible and attractive for the microbiome, but it is replaced by biogenic carbon formed from microbial necromass to a much higher extent.

The potential lever of such chemical technology is breathtaking: the optimal concentration of added A-HS of 0.03 wt % determined above corresponds to about 1 ton anthropogenic carbon per ha, the following growth of the living matter soil system however binds between 10 t and 70 t C per ha by self-processes, mostly employing in the absence of plants soil photocatalysis as the primary step. If we multiply this with the 1.6 Gha arable land worldwide (see above), we are absolutely on the correct scale for a climate relevant carbon sequestration.

The observation that they are potentially not the same carbon atoms stored in soil over a thousand years is only a legal problem for a potential accounting of carbon credits, while science is used to value dynamic equilibria of carbon stored on the system level, as for instance it is done with every forest in equilibrium.

## 5 The Visions of Urban Farming and Cities Employing Autonomous Living Matter Systems

**Figure 12:**
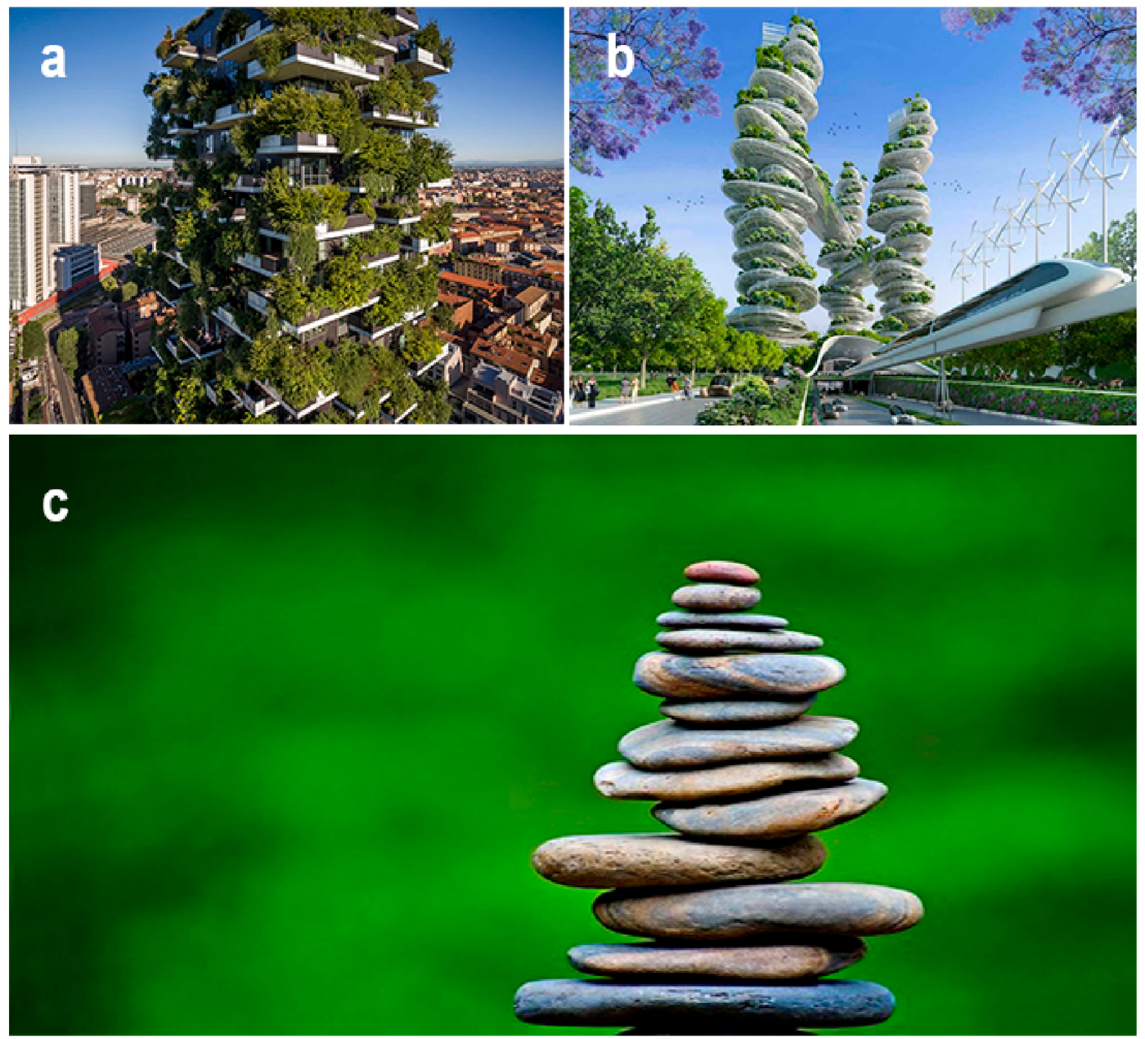
(a) Bosco Verticale in Milano and (b) “Asian Cairns” by Vincent Callebaut (2013), the vision a “Farmscraper” for Shenzhen (c) its original “bionic” inspiration (picture rights by Shutterstock under Creative Commons licenses).

Although this last potential application is more a joint architectural and cultural vision than reality, we want to come back to the starting point of living engineered materials for improving also the technology and life quality in inner cities. Greening skyscrapers and facades or even just planting inner city trees improves air quality, moderate temperature peaks, improves thermal insulation of buildings by an active, responding system. Modern pioneers of this movement are for instance the creators of Bosco Verticale (2014, Milano, Figure 11a), the already half-realized vision of a “Garden City Singapore” (starting in 1967), or the current plannings within Saudi Arabia’s Vision 2030 program. Just viewing Figure 12, we can anticipate a necessity of urban soil materials in largest amounts and then even with added future chemical and engineering performance profiles. Vertical façade greening for instance depends on a mechanical support structure, e.g., textile fabrics or biofoams, which then support or complement the mineral soil. Water binding in such situations is of special importance, but can be included in the engineering tasks of the then to-be-modified living matter system “green façade”. Urban farming integrated in building structures is another part of this enabled lifestyle, and this is closer to the science of how anthropogenic soils including active microbia can minimize fertilizer demands and establish a healthy microbiome, making vegetable plants more resistant and more nutritious than only by hydroponics.

## 6 Summary and Abstraction

In this review, we described how minute amounts of amphiphilic polymers, humins, turn granular soil into a matrix to support and organize microbial life. It is a multiply described observation that soil fertility goes over a wide range strictly with the total carbon content of soil, that is, in the absence of organic matter life is not sustainably supported. Within the novel concept of “living engineering material hybrids”, we were able to put this role of humins to many physicochemical effects, such as water and ion binding, changing soil texture, and morphological changes of minerals, but the key is the support of organized microbial life. It is not only providing growth conditions and food for the single microbial species, it is moreover the enabling of complex interaction patterns between the single species to enable effective social communities then being able to fulfill also complex tasks, such as “soil fertilility”. In simple words, we can state that many biological processes are aligned along proton–electron transfer (such as all oxidations, reductions, CO_2_ binding, N_2_ fixation, methane metabolization, etc..), and humins are simply very effective proton and electron buffers, a “microbial banking system” for proton–electron exchange, or in the language of chemistry, a mediator.

The availability of anthropogenic humic substances (A-HS) made from biomass leftovers now allows testing of complex hypotheses by chemical variation of the A-HS, i.e., AHS becomes now a reproducible, experimentally standardized, and variable product. Most previous research relied on the comparison of a polytype of very diverse products extracted from different soils in terms of, e.g., region, climate conditions, and extraction process.

In terms of the “living materials system”, the natural (and now synthetic) role model of soil might provide lessons also for the engineering of other living materials: providing nutrients and an environment with appropriate structure and mechanical properties is important, but sub per mille amounts of mediators seem to be very beneficial, too.

Such self-organized, self-supported, and sustainable living engineering materials indeed then can fulfill a number of important engineering tasks, such as in agriculture, environmental remediation, architecture, and heat management, but also in health, art, and fashion. It is a not too brave a prediction that our future cities and even social life will not do without.

## Abbreviations

A-HS: artificial humic substances
A-HA: artificial humic acid
A-FA: artificial fulvic acid
TOC: total organic carbon
DOC: dissolved organic carbon
SOM: soil organic matter
HTC: hydrothermal carbonization
HTH: hydrothermal humification
BC: biochar
FI: fluorescence index
BIX: biological index
